# Immune Resistance in Glioblastoma: Understanding the Barriers to ICI and CAR-T Cell Therapy

**DOI:** 10.3390/cancers17030462

**Published:** 2025-01-29

**Authors:** Thomas Eckert, MS Zobaer, Jessie Boulos, Angela Alexander-Bryant, Tiffany G. Baker, Charlotte Rivers, Arabinda Das, William A. Vandergrift, Jaime Martinez, Alicia Zukas, Scott M. Lindhorst, Sunil Patel, Ben Strickland, Nathan C. Rowland

**Affiliations:** 1 School of Medicine, University of South Carolina, Columbia, SC 29209, USA; 2 MUSC Institute for Neuroscience Discovery (MIND), Medical University of South Carolina, Charleston, SC 29425, USA; zobaer@musc.edu (M.S.Z.); bakert@musc.edu (T.G.B.); rowlandn@musc.edu (N.C.R.); 3 Department of Neurosurgery, Medical University of South Carolina, Charleston, SC 29425, USA; dasa@musc.edu (A.D.); vandergr@musc.edu (W.A.V.); martinezj@musc.edu (J.M.); zukas@musc.edu (A.Z.); lindhors@musc.edu (S.M.L.); patels@musc.edu (S.P.); strickbe@musc.edu (B.S.); 4 Department of Bioengineering, Clemson University, Clemson, SC 29634, USA; jboulos@clemson.edu (J.B.); angelaa@clemson.edu (A.A.-B.); 5 Department of Pathology and Laboratory Medicine, Medical University of South Carolina, Charleston, SC 29425, USA; 6 Department of Radiation Oncology, Medical University of South Carolina, Charleston, SC 29425, USA; iveyc@musc.edu; 7 Hollings Cancer Center, Medical University of South Carolina, Charleston, SC 29425, USA

**Keywords:** neuroimmunology, glioblastoma, immune microenvironment, T cells

## Abstract

Background: Glioblastoma (GBM) is the most common primary malignant brain tumor, with fewer than 5% of patients surviving five years after diagnosis. The introduction of immune checkpoint inhibitors (ICIs), followed by chimeric antigen receptor (CAR) T-cell therapy, marked major advancements in oncology. Despite demonstrating efficacy in other blood and solid cancers, these therapies have yielded limited success in clinical trials for both newly diagnosed and recurrent GBM. A deeper understanding of GBM’s resistance to immunotherapy is essential for enhancing treatment responses and translating results seen in other cancer models. Objectives: In this review, we examine clinical trial outcomes involving ICIs and CAR-T for GBM patients and explore the evasive mechanisms of GBM and the tumor microenvironment. Findings and Discussion: Multiple clinical trials investigating ICIs in GBM have shown poor outcomes, with no significant improvement in progression-free survival (PFS) or overall survival (OS). Results from smaller case studies with CAR-T therapy have warranted further investigation. However, no large-scale trials or robust studies have yet established these immunotherapeutic approaches as definitive treatment strategies. Future research should shift focus from addressing the scarcity of functional T cells to exploiting the abundant myeloid-derived cells within the tumor microenvironment. Conclusions: Translating these therapies into effective treatments for glioblastoma in humans remains a significant challenge. The highly immunosuppressive nature of GBM and its tumor microenvironment continue to hinder the success of these innovative immunotherapeutic approaches. Targeting the myeloid-derived compartment may lead to more robust and sustained immune responses.

## Simple Summary

Immune checkpoint inhibitors (ICIs) and chimeric antigen receptor (CAR) T-cell therapy have been successful in treating select solid and blood borne cancers. However, ICIs and CAR-T have failed to yield benefit in glioblastoma (GBM) across multiple randomized trials. The major obstacles in ICI and CAR-T stem from the complex, immunosuppressive microenvironment, brain barrier penetrance, and systemic leukopenia inherent to GBM. ICIs and CAR-T therapy bolster the immune response by mitigating tumor-cell induced exhaustion through blocking critical checkpoint pathways and equipping cytotoxic T-cells with targetable tumor-specific proteins, respectively. This review highlights the clinical trials done using ICI and CAR-T therapy and the significant challenges that GBM and the immunosuppressive tumor microenvironment (TIME) pose for future strategies. Overcoming GBM’s profound immunosuppression may lie within the abundant myeloid-derived cell population in the microenvironment.

## 1. Introduction

Glioblastoma (GBM), the most common primary malignant brain tumor, remains a devastating diagnosis despite comprehensive treatment strategies with surgery, chemotherapy, and radiation [1,2,3,4]. Since the introduction of the Stupp regimen decades ago, there have been limited advancements in GBM treatment, leaving patients with an overall survival of just 15 months and a 5-year survival rate of less than 5% [2,3,5]. The tumor immunosuppressive microenvironment (TIME) supporting GBM is poorly understood and highly complex. Nevertheless, it has emerged as a key target for immunotherapy and remains one of the biggest limitations in improving patient outcomes [6,7,8,9,10,11]. While numerous pathways within the TIME are potential targets, the PD-1/PD-L1 and CTLA-4 pathways have garnered significant recent interest. These two immune checkpoint pathways have been extensively studied and tested in preclinical and clinical trials [10,11]. Immune system checkpoints are essential for maintaining a balance between T-cell activation and inhibition, preventing autoimmunity, and regulating the anti-tumor immune response. In GBM, tumor cells exploit these checkpoints to suppress anti-tumor immunity, resulting in an accumulation of exhausted and inactive T lymphocytes within the tumor microenvironment [12]. The blockade of these pathways with immune checkpoint inhibitors (ICIs) revealed promising results in preclinical studies; however, recent clinical trials in humans have not translated similar findings [13,14,15]. Complementing this approach, chimeric antigen receptor (CAR)-T cell therapy has emerged as an alternative therapeutic strategy. Re-engineering T cells with synthetic CARs allows them to specifically recognize and destroy cancer cells expressing tumor-specific antigens, empowering them to mount a more potent and targeted attack against the tumor [16]. ICIs and CAR-T cell therapies have shown success in treating hematological malignancies and some solid tumors, earning FDA approval. However, their efficacy in GBM remains limited due to its low mutational burden, inter- and intra-tumor heterogeneity, low T-cell infiltration, and the TIME [12,17,18]. Multiple randomized controlled trials (RCTs) with ICIs have failed to improve outcomes in GBM, and only one completed trial suggests limited safety and efficacy for CAR-T cell monotherapy. This review examines the current literature and clinical trials on ICIs and CAR-T cell therapies in GBM, focusing on its unique characteristics of the tumor microenvironment and detailing the PD-1/PD-L1, CTLA-4, and CAR-T pathways. While numerous reviews and studies focus on the limitations and failures of ICIs and CAR-T cell therapies, this review suggests exploring a different avenue within the microenvironment [4,7,19,20]. Today’s current strategies target the pathways driving the depletion and exhaustion of T lymphocytes within the TIME. However, emerging evidence suggests that future breakthroughs may be within an alternative immune cell population. While the TIME and direct effects from tumor cells have traditionally been implicated in the exhaustion of T lymphocytes, recent studies highlight the critical role of myeloid-derived cells, particularly tumor-associated macrophages (TAMs) and myeloid-derived suppressor cells (MDSCs), in exacerbating T-cell exhaustion [21,22]. Targeting TAMs and other myeloid derivatives may revert T-cell exhaustion and unlock more effective and durable outcomes in GBM immunotherapy.

## 2. PD-1/PD-L1

**Figure 1:**
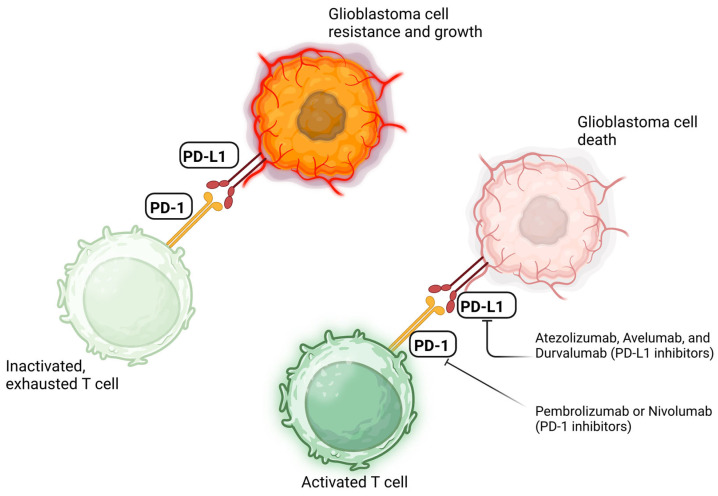
Schematic diagram illustrating how PD-1 and PD-L1 inhibitors prevent binding to their respective substrates, allowing the T cell to remain “on” and active in the fight against tumor cells. Created in BioRender. Suresh, R. (2025) https://BioRender.com/k07i775 (accessed on 18 January 2025).

First discovered in 1992 by Ishida et al., the PD-1/PD-L1 pathway has long been analyzed and targeted for the treatment of various autoimmune, infectious, transplant, and neoplastic pathologies [23,24,25]. Programmed cell death protein 1 (PD-1) and programmed cell death ligand 1 (PD-L1) are proteins with immunosuppressive capabilities mainly expressed on activated T cells and antigen-presenting cells (APCs), including tumor cells, respectively [24,25]. Higher PD-L1 expression in GBM has been associated with poorer survival outcomes, as PD-L1 on tumor cells binds to PD-1 on activated T lymphocytes. This interaction renders the T cells inactive, leading to an inadequate immune response and a population of anergic helper and cytotoxic T lymphocytes within the microenvironment [26,27,28]. The creation of novel antibodies that block the PD-1/PD-L1 pathway has helped to preserve the cytotoxic, anti-tumor state in T lymphocytes (Figure 1) [29].

## 3. CTLA-4

**Figure 2:**
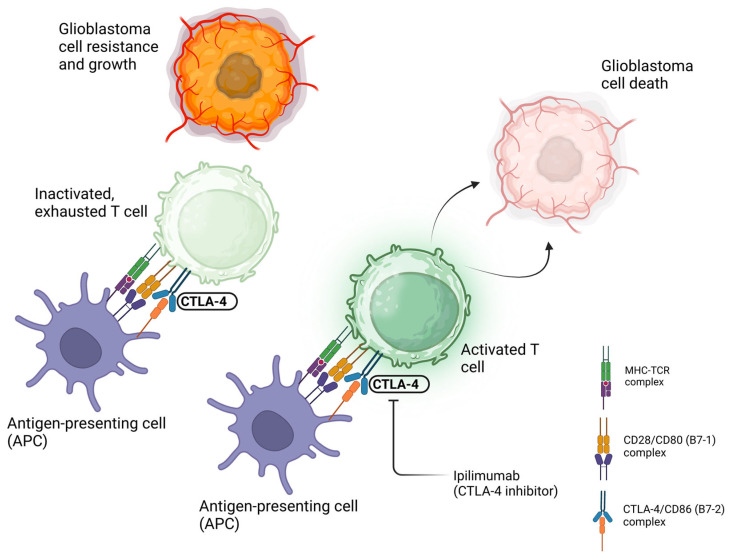
Schematic diagram depicting the interaction between CTLA-4 and CD80, which delivers inhibitory signals that downregulate the T-cell immune response; thus, an anti-CTLA-4 antibody prevents this binding and keeps the T cell in a proactive, anti-tumor state. Created in BioRender. Suresh, R. (2025) https://BioRender.com/l15c497 (accessed on 18 January 2025).

Cytotoxic T-lymphocyte antigen 4 (CTLA-4), a surface receptor expressed on T cells, competes with CD28 for binding to costimulatory molecules (CD80 and CD86) on APCs [29]. CTLA-4 binds to CD80 and CD86 with a higher affinity and avidity than CD28 and inhibits the stimulatory, CD28-mediated signal needed for T-cell activation [29]. GBM cells exploit this property of CTLA-4 by inducing the differentiation of T cells into regulatory T cells (Tregs), which have a higher surface expression of CTLA-4. This, in turn, reduces the activation of surrounding cytotoxic T lymphocytes and weakens the anti-tumor response [29]. By blocking the binding of CTLA-4 and CD80, anti-CTLA-4 antibodies can reactivate the function of tumor-infiltrating T cells (Figure 2).

## 4. Translating ICI Therapy for GBM

Since the FDA approval of ipilimumab (anti-CTLA-4) for the treatment of metastatic melanoma in 2011, five additional inhibitors of the PD-1/PD-L1 pathway have been approved for multiple cancers [17]. These novel drugs have provided oncologists with a new tool to harness the host immune system, preventing immune tolerance and reducing T-cell exhaustion in various malignancies [30]. Given their functions, combination therapy with anti-PD-1, anti-PD-L1, and anti-CTLA-4 inhibitors has become an intriguing approach to cancer treatment. Consequently, ipilimumab (anti-CTLA-4) and nivolumab (anti-PD-1) were approved as a combination therapy for the treatment of melanoma in 2017 [17]. Anti-CTLA-4 therapy enhances the antigen-specific T cell-dependent immune response, while anti-PD-1/PD-L1 activates the ability of cytotoxic T cells to lyse cancer cells, resulting in mutually synergistic effects when delivered together [31]. The various clinical trials and their respective outcomes involving ICI as a monotherapy, combination therapy, or as neoadjuvant therapy are discussed below and depicted in Table 1.

### 4.1. Single ICI Therapy

The efficacy of immune checkpoint inhibition, specifically PD-1 inhibition, has been evaluated in several clinical trials, both as monotherapy and in combination with standard care.

CheckMate 143, an open-label, randomized, phase III trial, examined the efficacy of nivolumab (anti-PD-1) against bevacizumab, an anti-vascular endothelial growth factor (VEGF) antibody approved by the FDA in 2009 for recurrent GBM [32,45]. This study included 369 patients with WHO grade 4 recurrent glioblastoma who previously received standard-of-care therapy. Patients were randomized to either receive nivolumab (3 mg/kg) or bevacizumab (10 mg/kg) every two weeks until disease progression, adverse events, or death [32]. Therefore, 182 patients were assigned to the nivolumab arm and 165 to the bevacizumab arm. After a median follow-up of 9.5 months, median overall survival (mOS) was similar for both groups: 9.8 months (95% CI, 8.2–11.8) for nivolumab arm versus 10.0 months (95% CI, 9.0–11.8) for bevacizumab arm. The 12-month overall survival (OS) rate was 42% for both groups [32]. However, bevacizumab showed superior progression-free survival (PFS), with a median PFS of 3.5 months (95% CI, 2.9–4.6) compared to 1.5 months (95% CI, 1.5–1.6) for nivolumab (*p* < 0.001). Overall, this study demonstrated no significant improvement in the primary endpoint of overall survival when comparing immune checkpoint inhibition to anti-VEGF therapy [32].

The CheckMate 498 trial compared the efficacy of nivolumab with radiation therapy against TMZ and radiation therapy (Stupp regimen) in patients with newly diagnosed MGMT-unmethylated GBM [33]. In this phase III trial, 560 patients were randomized to receive either standard radiotherapy (60 Gy) combined with nivolumab (240 mg every 2 weeks for 8 cycles, then 480 mg every 4 weeks) or radiation therapy with TMZ (75 mg/m^2^ daily during radiation therapy and 150–200 mg/m^2^/day for 5 days every 28-day cycle during maintenance). Each treatment arm included 280 patients [33]. The control arm that received radiation therapy with TMZ demonstrated longer mOS of 14.9 months (95% CI, 13.3–16.1) and higher 24-month OS of 21.2% (95% CI, 16.4–26.5) compared to the nivolumab arm with mOS of 13.4 months (95%, 12.6–14.3) and 24-month OS rate of 10.3% (95%CI, 6.8–14.6) [33]. These findings demonstrated that standard-of-care therapy with TMZ is superior to radiation therapy and nivolumab in patients with *MGMT*-unmethylated GBM [33].

CheckMate 548 took the next step of incorporating nivolumab with standard-of-care therapy [14]. In this phase III trial, 716 patients with newly diagnosed *MGMT*-methylated GBM were randomized to receive RT and TMZ with either nivolumab or placebo. Of these, 709 patients received the allocated treatment (355 in the nivolumab arm and 354 in the placebo arm). The experimental arm had mOS, 12-month OS rate, and 24-month OS rates of 28.9 months (95% CI, 24.4–31.6), 82.7% (95% CI, 78.3–86.3), and 60.9% (95% CI, 54.4–66.8) [14]. The placebo arm had mOS of 32.1 months (95% CI, 29.4–33.8), 12-month OS rate of 87.7% (95% CI, 83.8–90.8), and 24-month OS rate of 67.1% (95% CI, 61.0–72.6) [14]. Historical studies of radiation therapy and TMZ in newly diagnosed *MGMT*-methylated GBM have reported mOS ranging from 21.4 to 26.3 months, with confidence intervals spanning 17.4–34.7 months [46,47,48]. Sub-analysis of patients with high PD-L1 expression (≥5%) suggested the potential for long-term survival benefits in this subgroup. While the addition of nivolumab did not result in statistically significant improvements in mOS, the subgroup of patients with long-term survival and high PD-L1 expression warrant further investigation [14].

### 4.2. ICI as Neoadjuvant Therapy

To evaluate the feasibility, safety, and effects of PD-1 inhibition as a neoadjuvant therapy, Schalper et al. conducted a single-arm phase II clinical trial administering nivolumab both pre- and post-surgery [34]. The treatment regimen included a single presurgical dose of nivolumab (3 mg/kg) given two weeks before surgery, followed by postsurgical doses every two weeks until disease progression or unacceptable toxicity occurred [34]. For the cohort, the median progression-free survival (mPFS) was 4.1 months, and the median overall survival (mOS) was 7.3 months. Although no significant improvements in these primary outcomes were observed, two patients who underwent gross total resection remained disease-free for 33.3 and 28.5 months, respectively. Analysis of tumor tissue samples collected before neoadjuvant nivolumab and after surgical resection revealed increased T-cell infiltration and T-cell receptor diversification, confirming a potential immunomodulatory effect of the therapy. These outliers and the immunomodulatory effect support further investigation into the use of immune checkpoint inhibitors in the neoadjuvant setting [34].

In an open-label, single-center, single-arm phase trial involving 15 patients with recurrent glioblastoma (GBM), de Groot et al. investigated the effects of neoadjuvant pembrolizumab, a PD-1 inhibitor [35]. Eligible patients demonstrated MRI evidence of recurrence, defined as progressive or new contrast enhancement following initial treatment with the Stupp regimen. Participants received up to two doses of pembrolizumab prior to surgical resection on days 21 and 1, followed by additional doses every three weeks until disease progression or unacceptable toxicity [35]. The median progression-free survival (mPFS) was 4.5 months (95% CI, 2.27–6.83), with a 6-month PFS rate of 40% (95% CI, 17–63%). The median overall survival (mOS) was 20.3 months (95% CI, 8.64–28.45), and the estimated 1-year OS rate was 63% (95% CI, 32–83%). Immune analysis conducted by de Groot et al. reaffirmed the “cold” microenvironment characteristic of GBM, marked by low levels of CD4+ and CD8+ T lymphocytes and high concentrations of CD68+ macrophages, even after pembrolizumab treatment. These macrophages predominantly exhibited an immunosuppressive M2 phenotype. These findings underscore the formidable immune resistance exerted by GBM within its microenvironment and highlight the challenges of achieving effective immune modulation in this malignancy [35].

### 4.3. Multiple ICI Combination Therapy

The PD-1/PD-L1 and CTLA-4 pathways play distinct roles in T-cell activation and infiltration, operating at different stages of the immune response. This non-redundancy provides a compelling rationale for combining these therapies to achieve complementary or potentially synergistic effects, as highlighted by Khair et al. [49]. The success of combining multiple ICIs in other cancers, such as melanoma, has demonstrated promising outcomes and serves as the basis for exploring similar combinations in challenging malignancies like GBM [49].

To test this hypothesis, multiple clinical trials have investigated the combination of PD-1 and CTLA-4 inhibitors in patients with newly diagnosed and recurrent GBM. The NCT02794883 trial evaluated the efficacy of tremelimumab (anti-CTLA-4), durvalumab (anti-PDL1), and their combination in recurrent GBM [37]. Across the three arms–tremelimumab only, durvalumab only and combination therapy–mOS was 7.23 months (95% CI, 2.75–16.32), 11.71 months (95%CI, 8.33–32.71), and 7.70 months (95%CI, 7.41–40.14), respectively. The median PFS was similarly modest at 2.75 months (95% CI, 2.68–8.73), 4.36 months (95% CI, 2.94–32.74), and 4.91 months (95% CI, 2.91–120.40), indicating no significant improvement in outcomes [37]. Another trial, NCT03367715, investigated a combination of nivolumab (anti-PD-1), ipilimumab (anti-CTLA-4), and short-course radiation therapy in newly diagnosed *MGMT*-unmethylated GBM [41]. This single-arm phase II study reported a 1-year OS rate of 80% among the 10 patients, with a median OS of 16.85 months (95% CI, 4.49–32.89) and a median PFS of 5.92 months (95% CI, 1.48–13.93) [41]. The NCT04396860 phase II/III trial compared standard-of-care therapy versus radiation therapy combined with ipilimumab (anti-CTLA-4) and nivolumab (anti-PD-1) in patients with newly diagnosed *MGMT*-unmethylated GBM [36]. This study evaluated whether this combination could extend PFS (Phase II) and OS (Phase III) compared to standard-of-care therapy [36]. Preliminary results demonstrated no improvement in PFS, with a median PFS of 8.5 months and 7.7 months in the control and experimental arms, respectively, leading to discontinuation before Phase III [36]. Similarly, the NCT04145115 phase II trial, which evaluated the same combination of ipilimumab and nivolumab in recurrent GBM with high tumor mutational burden (TMB), has been suspended [15]. The rationale behind targeting GBM with high TMB stems from findings by Samstein et al., who found that high TMB was associated with improved overall survival in patients receiving ICI therapy [50]. In a cohort of 1662 patients, primarily with stage IV or metastatic melanoma, renal cell carcinoma, bladder carcinoma, or head and neck cancers, higher TMB was linked to improved overall survival, highlighting its potential as a predictor of ICI response [50]. With low TMB defined as 10 mutations/14.4 MB or less and high TMB defined as more than 20 mutations/14.4 MB, Hodges et al. demonstrated that 85.3% of gliomas (279 of 327) had low TMB [51]. Further analysis revealed that GBM, specifically, harbors a low mutational burden, with only 3.5% exhibiting high TMB [51]. In addition, Hodges et al. revealed that, in the GBMs with high TMB, there was decreased infiltration of cytotoxic, CD8+ T lymphocytes and PD-1 expressing T cells. These findings suggested that only a small subset of patients with GBM may benefit from ICI therapy and that low TMB is paradoxically associated with longer survival, contrary to the findings from Samstein et al. [51]. Together, these two studies underscore the complexity of translating findings from other cancers and a unique characteristic of GMB regarding TMB that is unlike other solid tumors.

### 4.4. Combination with Vaccine-Based and Viral Therapies

Active immunotherapy enhances the patient’s immune system by exposing it to antigens, and recent vaccine-based treatments for GBM have demonstrated potential in Phase I and II clinical trials [52]. In these therapies, patients receive vaccines containing tumor antigens, which are recognized by the adaptive immune system to elicit a targeted response against the tumor [52,53]. The delivery of GBM vaccines often involves peptide-based vaccines, which are ultimately presented to T cells by dendritic cells (DCs). In this approach, DCs are exposed to tumor antigens and mature ex vivo before being administered to the patient. Once administered, DCs travel to lymphoid tissues, where they present the tumor-specific antigen to resident T lymphocytes, subsequently activating and generating an immune response [53]. Oncolytic virotherapy is another recent promising and innovative approach. Oncolytic viruses (OVs) are genetically engineered, attenuated viruses designed to selectively target and destroy tumor cells. These viruses eliminate tumor cells and amplify the immune response both directly, through cell lysis following viral replication, and indirectly, by triggering the infiltration and activation of nearby T cells, DCs, and others via the release of tumor-specific antigens [54]. These approaches aim to reprogram and prime myeloid-derived cells in the tumor microenvironment with tumor-specific antigens. Vaccine-based and OV therapies have been extensively studied, with few studies showing promise and warranting further investigation [53,55,56].

Of interest are two clinical trials combining the use of viral or vaccine-based therapy with ICI therapy. The NCT04479241 phase II single-arm trial evaluated the safety and efficacy of PVSRIPO and pembrolizumab (anti-PD-1) in 25 patients with recurrent GBM [43]. The results from this study are much anticipated, as PVSRIPO, a live-attenuated poliovirus type 1 (Sabin) vaccine, has previously demonstrated tolerability. In a study of 61 patients with recurrent GBM, those who received PVSRIPO achieved 21% OS rate at the 24-month follow-up, with no reports of viral shedding or reactivation [56]. Another phase II study, NCT04013672, assessed the efficacy of pembrolizumab (anti-PD-1) with SurVaxM, a peptide vaccine targeted to a molecule produced by cancers called survivin [44]. In the 41 patients who received this combination, 34.5% (95%CI, 19.8–49.6) had no disease progression at 6 months following treatment, suggesting modest activity of this combination to improve PFS [44].

## 5. CAR T-Cell Therapy in GBM

Unlike ICIs, which work by neutralizing inhibitory signals that suppress immune responses after detection of cancer cells, CAR T-cell therapy enhances the ability of immune cells to specifically identify and target tumor cells [57]. Chimeric antigen receptors (CARs) are designed to help T cells recognize and target tumor cells with a specific antigen. The patient’s T cells are removed from the blood via leukapheresis, modified to express CARs, replicated, and reintroduced to trigger a long-lasting tumor-specific immune response [16]. CAR T-cell therapy has demonstrated efficacy in treating hematologic malignancies with anti-CD19 CAR-T therapy achieving FDA approval in 2017 for relapsed or refractory B-cell lymphoma [16]. Subsequent studies have shown that combining CAR T-cell therapy with ICIs can be effective in patients with relapsed B-cell acute lymphoblastic leukemia [58,59,60,61,62]. While CAR T-cell therapy has shown significant success in treating hematologic cancers, its use in GBM remains in the early stages, with ongoing clinical trials investigating its safety and efficacy against several common targets [63]. These targets have been identified as promising due to their overexpression on GBM cell surfaces and include B7-H3, EGFRvIII, and IL13Rα2. B7-H3 (CD276), an immune checkpoint molecule involved in tumor progression, along with receptors EGFRvIII and IL13Rα2, are highly overexpressed in GBM and associated with worse prognoses, making them attractive therapeutic targets [18,63]. Preclinical models have demonstrated promising initial results with these targets and have led to various clinical trials in humans (Table 2) [18,63].

Several case studies have prompted further investigations with clinical trials. In 2016, a 50-year-old patient with recurrent GBM experienced rapid disease progression and development of leptomeningeal disease involving both cerebral hemispheres while undergoing an investigative trial [83]. After discontinuation, the patient enrolled in another study and received CAR T-cells targeted to interleukin-13 receptor alpha 2 (IL13Rα2) [84]. Following resection of three progressive lesions, cycles of CAR-T therapy began with an initial infusion of 2 × 10^6^ cells followed by five infusions of 10 × 10^6^ cells into the resected tumor cavity and ventricular system [84]. Over 220 days, the patient received weekly infusions into the largest resected cavity and 10 additional intraventricular treatments, resulting in 77–100% reduction in size of all intracranial and spinal tumors [84]. This dramatic clinical response was sustained for 228 days until disease recurrence was noted with four distinct new lesions [83]. Another case report in 2021 highlighted a 59-year-old patient with *IDH1*-wildtype recurrent GBM who received CAR T-cells targeted to epidermal growth factor receptor variant III (CAR T-*EGFRvIII*) [84]. This patient received a single, peripheral infusion of 9.2 × 10^7^ CAR T-*EGFRvIII* cells after completing 3 months of standard-of-care therapy [85]. Therefore, 104 days after initiating CAR-T therapy, the patient underwent a second craniotomy for disease recurrence. The patient ultimately survived 36 months post-recurrence, surpassing typically reported survival outcomes with significant tumor regression. Additionally, CAR T-*EGFRvIII* cells persisted in the patient’s circulation for 29 months, marking the longest persistence reported in the literature [84]. Post-infusion histopathologic analysis of resected tumor tissue revealed reduced expression of IL13Rα2 and *EGFRvIII* in both patients, respectively [83,84]. These findings suggest that tumor recurrence and subsequent death may have resulted from a loss of response to CAR T-cell therapy due to diminished target expression. These cases underscore the potential of CAR T-cell therapies for GBM but emphasize the need for large-scale studies to optimize and evaluate their broader efficacy.

One of the only completed trials, from Goff et al., evaluated the safety of administering CAR T-*EGFRvIII* cells and determined subsequent six-month PFS [82]. The study was conducted using a Phase I/II design and included 18 patients with histologically proven glioblastoma expressing the *EGFRvIII* mutation. All patients received prior standard treatment with radiotherapy and chemotherapy. After determining the tolerability, the study proceeded to phase II with two cohorts: patients receiving steroids at the time of treatment and those not receiving steroids [82]. Eighteen patients enrolled and received infusion doses ranging from 6.3 × 10^6^ to 2.6 × 10^10^ cells. Secondary outcomes, mPFS and mOS, were modest at 1.3 months and 6.9 months, respectively [82]. The durability of the CAR T-cells correlated with larger dosing; however, significant hypoxia occurred in two patients at the highest dose with one resulting in a treatment-related death [82]. This phase I pilot trial showed that anti-EGFRvIII CAR T-cells provide no clinical benefit and had expected and manageable side effects, except for severe respiratory complications. Another phase I trial (NT03726515) was conducted to evaluate the safety of CAR T-EGFRvIII therapy combined with pembrolizumab (anti-PD-1) in patients with newly diagnosed EGFRvIII+ GBM (Table 3) [85]. The trial reported no dose-limiting toxicities, confirming the combination’s safety as the primary outcome. Secondary outcomes included a median PFS of 5.2 months (90% CI, 2.9–6.0 months) and a median OS of 11.8 months (90% CI, 9.2–14.2 months). On further analyses, this study revealed increased populations of exhausted, regulatory T cells within the tumor microenvironment, demonstrating safety, modest efficacy, and tumor microenvironment modulation [85].

In early 2024, Choi et al. reported results from three patients with recurrent GBM treated with CARv3-TEAM-E T-cells targeting epidermal growth factor receptor (*EGFR*) wild-type and epidermal growth factor receptor variant III (*EGFRvIII*) [86]. Rapid radiographic tumor regression, although transient in two of the three participants, was noted within days after receiving a single intraventricular infusion [86]. Notably, the CAR T-cells demonstrated anti-tumor activity even in the third patient, who did not have *EGFRvIII* expression, revealing the potential efficacy of this treatment in patients without either *EGFRvIII* expression or *EGFR* amplification. This study provides evidence that CAR T-cell therapy is an effective strategy for targeting multiple related antigens on glioblastoma cells and that *EGFR* is a viable immunotherapeutic target. The eventual tumor progression seen in the two patients corresponded with limited persistence of the CAR T-cells over weeks after the infusion [86]. Cytopathological analysis of the CSF samples in the three patients revealed temporary persistence and then exponential decrease in the first week after infusion. These findings support further investigation of engineered CAR T-cells in combination with strategies designed to enhance their persistence. One such strategy involves the combination with immune checkpoint inhibitors [86]. These studies aimed to evaluate safety, efficacy, tolerability, and overall survival, and they reflect the growing, early-stage research in CAR T-cell therapy for recurrent GBM. However, the challenges posed by GBM’s immune resistance mechanisms have limited the success of these trials.

## 6. GBM Resistance, Immune Evasion, and Challenges of Assessing Responses

GBM remains highly resistant to treatment due to significant inter- and intra-tumoral heterogeneity, systemic and local immunosuppressive mechanisms, and disruption of the blood–brain barrier (BBB) [87].

One of the most formidable challenges in treating glioblastoma (GBM) is its profound inter- and intra-tumoral heterogeneity. Inter-tumoral heterogeneity refers to variations in molecular and genetic profiles between different regions of a single tumor, while intra-tumoral heterogeneity refers to diverse genetic and molecular characteristics among individual cancer cells within the same tumor. This complexity significantly limits the efficacy of immunotherapies, as varying subpopulations of cells and tumor regions express different antigens and mutations [87]. In relation to molecular and genetic heterogeneity, tumor mutational burden (TMB) refers to the total number of mutations per million bases and has been discovered to be a prognostic indicator for ICI treatment response in solid tumors such as melanoma and non-small-cell lung cancer [88]. These other solid tumors demonstrate high TMB and a response to ICI that is not translated to GBM. By contrast, a high TMB in gliomas has not been reported to correlate with better outcomes and response to ICI therapy [89]. Consequently, ICI and CAR T-cell therapies, which rely on targeting specific pathways or antigens, often fall short of providing comprehensive and durable responses. Addressing the mutational burden and heterogeneity remains a critical hurdle in advancing effective GBM treatments [87].

GBM exerts profound immunosuppressive effects, both systemically and within the tumor microenvironment, undermining the anti-tumor immune response. Notably, the depletion of T cells in GBM patients occurs independently of leukopenia commonly induced by radiation therapy and chemotherapy [59,90]. Systemically, GBM depletes T cells in both number and function, a phenomenon partially attributed to the tumor-induced loss of sphingosine-1-phosphate receptor 1 (S1PR1). This disruption impairs lymphocyte trafficking, sequestering naive T cells in the bone marrow and contributing to persistent T cell lymphopenia, a hallmark of GBM that has remained incompletely understood [59,90]. The S1P–S1PR1 axis, crucial for lymphocyte trafficking, is increasingly recognized as a key player in this process. At the microenvironmental level, GBM manipulates immune responses through the secretion of immunosuppressive cytokines and the expression of inhibitory ligands. For instance, interleukin-6 (IL-6), programmed death ligand-1 (PD-L1), and indoleamine 2,3-dioxygenase (IDO) collectively promote the recruitment and expansion of regulatory T-cells (Tregs) while suppressing the proliferation and activity of cytotoxic CD8+ T lymphocytes [59]. This results in sparse cytotoxic T-cell infiltration at the tumor site and, along with the profound heterogeneity of the tumor, leads to T-cell exhaustion and anergy [59,91]. Depleted and exhausted T lymphocytes are incapable of generating a robust immune response, which is further limited by the need for major histocompatibility complex (MHC) protein expression. In the tumor microenvironment, microglia play a crucial role in presenting antigens via MHC class I to activate cytotoxic CD8+ T lymphocytes. However, GBM downregulates MHC expression through the release of immunosuppressive cytokines such as IL-10 and TGF-β, preventing the activation of tumor-infiltrating lymphocytes via antigen presentation from microglia and myeloid-derived cells [92]. Together, these systemic and localized immune evasion strategies contribute to the formidable resistance of GBM to immunotherapies.

GBM’s resistance to therapies, especially CAR-T cell therapy, is also influenced by the physical barrier of the blood–brain barrier (BBB). For CAR-T therapy to be effective, the engineered T cells must consistently cross the BBB and reach the central nervous system (CNS) [87]. GBM distorts the BBB by disrupting the normal contact between endothelial cells and the basement membrane, limiting the trafficking of CAR T-cells and other drugs into the tumor site [87,91]. However, the tumor’s extensive angiogenesis and rapid proliferation result in a disorganized and abnormal vasculature, which creates significant obstacles for the efficient distribution and diffusion of CAR T-cells and other drugs [87]. The irregular blood vessel network impairs the ability of CAR T-cells to adequately infiltrate the tumor, limiting their therapeutic effectiveness and contributing to the challenge of achieving meaningful treatment responses. The rapid growth of tumor cells exacerbates the hypoxia within the microenvironment, triggering a continuous release of angiogenic factors to provide the tumor cells with adequate blood and oxygen [29,59]. These hypoxic conditions, along with the recruitment of immunosuppressive Tregs and TAMs, lead to a continuous cycle of angiogenesis and tumor resistance, making it difficult for ICIs and CAR-T therapies to act on or infiltrate the TME [29].

In addition to the numerous immunosuppressive mechanisms employed by GBM, clinicians face the added challenge of accurately assessing responses to immunotherapy, further complicating the management of patients with this aggressive tumor. The iRANO criteria was developed as an adaptation of the RANO guidelines to better assess responses to specifically immunotherapy by providing standardized metrics across clinical trials and routine practice [93]. The unique characteristics of GBM, such as inter- and intra-tumoral heterogeneity, combined with the challenge of distinguishing true tumor progression from pseudoprogression (PD), complicates the response assessment. PD occurs in approximately 10–20% of newly diagnosed GBM patients after standard-of-care therapy with TMZ or TMZ and radiation therapy, typically within three months [93]. In contrast, the timeline for PD associated with immunotherapy remains poorly defined. The differentiation between PD and true progression is critical, as early radiographic changes that indicate progression may not rule out the patient benefiting from immunotherapy in the future. These radiographic changes can include increased enhancement and edema, mimicking the inflammation commonly secondary to immunotherapies [93]. This creates a dilemma for clinicians, who must balance allowing sufficient time for a potential immunotherapeutic response against the risk of delaying intervention for true disease progression. The updated iRANO guidelines aim to refine the interpretation of early progressive imaging changes, allowing clinicians to focus on minimizing premature termination of therapy and maximizing patient care and safety [93]. Multiple studies have investigated the use of deep learning and machine learning models to alleviate this challenge of discriminating PD and true progression for clinicians [94,95].

## 7. Conclusions and Future Directions—The Myeloid-Derived Compartment

The persistent resistance of GBM to treatment, particularly to ICIs and CAR T-cell therapy, is driven by a combination of complex factors including inter- and intra-tumoral heterogeneity, the TIME, and reduced drug trafficking across the blood–brain barrier. These collectively limit the efficacy of current immunotherapies and explain the disappointing results observed in clinical trials using these T-cell oriented therapies. Overcoming these challenges requires innovative strategies to improve the infiltration and persistence of T lymphocytes into the tumor microenvironment. Future research should pivot from solely addressing the scarcity of functional T cells to leveraging the abundance of myeloid-derived cells as therapeutic targets. TAMs and other myeloid derived cells are significantly more abundant than exhausted T cells within the microenvironment, and their presence is positively correlated with tumor size and inversely correlated with survival outcomes [96,97]. These cells promote tumor growth and persistence while simultaneously suppressing cytotoxic T lymphocyte activity, thereby reinforcing the immunosuppressive and pro-tumor landscape [21,22]. Within the TIME, myeloid cells, including TAMs and myeloid-derived suppressor cells (MDSCs), are converted into potent immunosuppressive cells. These MDSC’s lack mature myeloid cell markers, such as HLA-DR, a MHC class II molecule. Combined with GBM’s reduced MHC expression, MDSCs contribute to TAM differentiation into the pro-tumor M2 phenotype and subsequent impaired T-cell-mediated immune responses [97]. TAMs, rather than the tumor cells, are the primary source of antigenic exposure that drives the exhaustion of functional T lymphocytes. Studies have shown that TAM depletion reduces the rate of T-cell exhaustion and enhances the efficacy of ICIs, such as anti-PD-1 therapy [21]. Addressing the challenges of ICIs and CAR-T therapy in GBM may require the development of strategies to reeducate or deplete TAMs, target their interaction with T cells, or design therapeutics that modulate the myeloid-derived compartment to ultimately improve the persistence of cytotoxic T lymphocyte responses [21,22]. Future strategies should focus on the myeloid-derived compartment, thereby redefining the immunotherapeutic landscape.

## Figures and Tables

**Table 1:** Clinical trials combining single or multiple ICI therapy compared to standard-of-care therapy for glioblastoma. Information obtained from ClinicalTrials.gov, accessed on 16 January 2025.

ID	Duration	Authors	Phase	Enrollment	Target	Diagnosis	Treatment Arm	Control Arm	Outcomes	Recruitment Status
*NCT02017717 (CheckMate 143) [32]*	2014–2024	Reardon et al.	III	529	PD-1	Recurrent GBM (rGBM)	Nivolumab (anti-PD-1)	Bevacizumab (anti-VEGF)	No significant difference in overall survival (OS)	Completed
*NCT02617589 (CheckMate 498) [33]*	2016–2022	Omuro et al.	III	560	PD-1	Newly diagnosed GBM (nGBM)	Nivolumab + RT	RT + TMZ	No improvement in median overall survival (mOS) and progression-free survival (PFS)	Completed
*NCT02667587 (CheckMate 548) [14]*	2016–2024	Lim et al.	III	716	PD-1	nGBM	Nivolumab + RT + TMZ	RT + TMZ	No improvement in mOS and median PFS (mPFS)	Completed
*NCT02550249 [34]*	2015–2017	Schalper et al.	II	29	PD-1	nGBM + rGBM	Neoadjuvant nivolumab	Single arm	No improvement in mOS and mPFS	Completed
*NCT02337686 [35]*	2015-	de Groot et al.	II	18	PD-1	rGBM	Neoadjuvant pembrolizumab	Single arm	No improvement in mOS and mPFS	Active, not recruiting
*NCT04396860 [36]*	2020-	Lassman et al.	II/III	159	PD-1 + CTLA-4	nGBM + unmethylated MGMT	Nivolumab + ipilimumab + RT	RT + TMZ	No improvement in PFS	Active, not recruiting
*NCT04145115 [15]*	2020-	Dunn et al.	II	37	PD-1 + CTLA-4	rGBM + high mutational burden (TMB)	Nivolumab + ipilimumab	Single arm	No results posted	Suspended
*NCT02794883 [37]*	2016–2020	Raizer et al.	II	36	PD-L1 + CTLA-4	rGBM	Durvalumab + tremelimumab	Parallel assignment	No improvement in mOS and mPFS	Completed
*NCT03636477 [38]*	2018–2021	Gelb et al.	I	21	PD-1	rGBM	Nivolumab + Ad-RTS-hIL-12 + Veledimex	Single arm	Established safety; no improvement in OS and PFS	Completed
*NCT03661723 [39]*	2018–2024	Reardon et al.	II	60	PD-1	rGBM	Pembrolizumab + Bevacizumab + Reirradiation	Parallel assignment	No improvement in mPFS and mOS	Completed
*NCT02337491 [40]*	2015–2020	Reardon et al.	II	80	PD-1	rGBM	Pembrolizumab +/− Bevacizumab	Parallel assignment	No improvement in mOS and PFS at 6 months	Completed
*NCT03367715 [41]*	2018–2022	NYU Langone	II	10	PD-1 + CTLA-4	MGMT unmethylated nGBM	Nivolumab + ipilimumab + short-course RT	Single arm	No improvement in PFS and OS	Completed
*NCT04977375 [42]*	2021-	Patil et al.	I/II	10	PD-1	rGBM	Pembrolizumab + stereotactic radiosurgery + surgical resection	Single arm	No results posted	Recruiting
*NCT04479241 [43]*	2020–2024	Franklin et al.	II	25	PD-1	rGBM	Lerapolturev (PVSRIPO) + pembrolizumab	Single arm	No results posted	Completed
*NCT04013672 [44]*	2020–2022	Peereboom et al.	II	41	PD-1	rGBM	Pembrolizumab + SurVaxM	Single arm	No improvement of PFS at 6 months	Completed

**Table 2:** Current clinical trials exploring CAR T-cell therapy in GBM. Information obtained from ClinicalTrials.gov, accessed on 15 January 2025.

ID	Duration	Authors/Sponsor	Phase	Enrollment	Target/Therapy	Diagnosis	Outcomes	Status
*NCT05868083 [64]*	2022–2024	Shanghai Simnova Biotechnology	I	16	SNC-109 CAR-T cells	rGBM	No results posted	Recruiting
*NCT05627323 [65]*	2023–	Litten et al. Chimeric Therapeutics	I	42	CHM-1101 CAR-T cells	MMP2+ rGBM or progressive GBM (pGBM)	No results posted	Active, not recruiting
*NCT05241392 [66]*	2022–	Zhang et al. Beijing Tiantan Hospital	I	30	Anti-B7-H3 CAR-T cells	rGBM	No results posted	Active
*NCT06691308 [67]*	2024–	Cheng et al. Beijing Immunochina Medical Science & Technology	I	6	WL276 CAR-T cells	rGBM or pGBM	No results posted	Not yet recruiting
*NCT05577091 [68]*	2023–	Zhang et al. Beijing Tiantan Hospital	I	10	Tris-CAR-T cells	rGBM	No results posted	Recruiting
*NCT05353530 [69]*	2023	Ghiaseddin et al. University of Florida	I	18	IL-8 CD70 CAR-T cells	CD70+ newly diagnosed GBM (ndGBM)	No results posted	Recruiting
*NCT05366179 [70]*	2022–	Rauf et al. UNC Lineberger Comprehensive Cancer Center	I	36	B7-H3 CAR-T cells	rGBM	No results posted	Recruiting
*NCT04385173 [71]*	2022–	Zhejiang University	I	12	B7-H3 CAR-T cells	rGBM	No results posted	Unknown
*NCT06616727 [72]*	2023–	Shanghai Simnova Biotechnology	I	50	SNC-109 CAR-T cells	rGBM	No results posted	Enrolling by invitation
*NCT04077866 [73]*	2023–	Zhejiang University	I/II	40	B7-H3 CAR-T cells	rGBM	No results posted	Recruiting
*NCT03170141 [74]*	2020–	Chang et al. Shenzhen Geno-Immune Medical Institute	I	20	Autologous IgT cells	rGBM	No results posted	Enrolling by invitation
*NCT05660369 [75]*	2023–	Maus et al.	I	26	CARv3-TEAM-E T cells	rGBM or ndGBM	No results posted	Recruiting
*NCT06186401 [76]*	2024–	Okada et al. University of California San Francisco	I	20	Anti-EphA2/IL-13alpha2 CAR (E-SYNC) T cells	EGFRvIII+ ndGBM or rGBM	No results posted	Recruiting
*NCT04214392 [77]*	2020–	Badie et al. City of Hope Medical Center	I	36	CLTX-CAR T cells	MPP2+ rGBM or pGBM	No results posted	Recruiting
*NCT06482905 [78]*	2024–	Ji et al. Tcelltech inc.	I	52	B7-H3 CAR-T cells	rGBM or pGBM	No results posted	Not yet recruiting
*NCT05802693 [79]*	2023–	Yang et al. Beijing Tsinghua Chang Gung Hospital	I	22	EGFRvIII CAR-T cells	rGBM	No results posted	Not yet recruiting
*NCT04045847 [80]*	2019–	Xijing Hospital	I	31	Anti-CD147 CAR-T cells	rGBM	No results posted	Unknown
*NCT04003649 [81]*	2019–	Badie et al. City of Hope Medical Center	I	60	IL13Rα2-CAR T cells + ICIs (nivolumab and ipilimumab)	rGBM	No results posted	Recruiting
*NCT01454596 [82]*	2012–2019	Rosenberg et al. National Cancer Institute (NCI)	I/II	18	EGFRvIII CAR-T cells	rGBM	mOS 6.9 months, PFS 1.3 months	Completed

**Table 3:** Current clinical trials exploring CAR T-cell therapy in combination with ICI therapy in GBM. Information obtained from ClinicalTrials.gov, accessed on 15 January 2025.

ID	Duration	Authors	Phase	Enrollment	Target/Therapy	Diagnosis	Outcomes	Status
*NCT03726515 [85]*	2021–2023	Bagley et al.	I	7	EGFRvIII CAR-T cells + pembrolizumab (anti-PD-1)	ndGBM	mOS 11.8 months, mPFS 5.2 months	Completed

## Data Availability

No new data were created or analyzed in this study. Data sharing is not applicable to this article.
